# Cellulase hyper-production by *Trichoderma reesei* mutant SEU-7 on lactose

**DOI:** 10.1186/s13068-017-0915-9

**Published:** 2017-10-04

**Authors:** Chengcheng Li, Fengming Lin, Le Zhou, Lei Qin, Bingzhi Li, Zhihua Zhou, Mingjie Jin, Zhan Chen

**Affiliations:** 1 State Key Laboratory of Bioelectronics, School of Biological Science and Medical Engineering, Southeast University, Nanjing, 210096 China; 2 Key Laboratory of Systems Bioengineering (Ministry of Education), School of Chemical Engineering and Technology, Tianjin University, Weijin Road 92, Nankai District, Tianjin, 300072 People’s Republic of China; 3 Key Laboratory of Synthetic Biology, Institute of Plant Physiology and Ecology, Shanghai Institutes for Biological Sciences, Chinese Academy of Sciences, Shanghai, 200032 China; 4 School of Environmental and Biological Engineering, Nanjing University of Science and Technology, Nanjing, 210094 China; 5 Department of Chemistry, University of Michigan, 930 North University Avenue, Ann Arbor, MI 48109 USA; 6 35 Jinxianghe Road, Xuanwu District, Nanjing, 210008 Jiangsu Province China

**Keywords:** *Trichoderma reesei*, Soluble carbon source, Cellulase, Hemicellulase, β-Glucosidase, Hyper-production

## Abstract

**Background:**

The induction of cellulase production by insoluble carbon source cellulose was a common and efficient strategy, but has some drawbacks, such as difficult fermentation operation, substantial cellulase loss, long fermentation time, and high energy-consumption, resulting in high cost of cellulase production in industry. These drawbacks can be overcome if soluble carbon sources are utilized as the inducers for cellulase production. However, until now the induction efficiency of most soluble carbon sources, especially lactose and glucose, is still inferior to cellulose despite extensive efforts have been made by either optimizing the fermentation process or constructing the recombinant strains. Therefore, strain improvement by metabolic engineering for high induction efficiency of soluble carbon sources is of great interest.

**Results:**

*Trichoderma reesei* mutant SEU-7 was constructed from *T. reesei* RUT-C30 with the overexpression of endogenous gene β-glucosidase (BGL1) by insertional mutagenesis via *Agrobacterium tumefaciens*-mediated transformation (AMT). Compared to RUT-C30, SEU-7 displays substantially enhanced activities of both cellulase and hemicellulase when grown on either lactose or cellulose. The induction efficiency with lactose was found to be higher than cellulose in strain SEU-7. To the best of our knowledge, we achieved the highest FPase activity in SEU-7 in both batch culture (13.0 IU/mL) and fed-batch culture (47.0 IU/mL) on lactose. Moreover, SEU-7 displayed unrivaled pNPGase activity on lactose in both batch culture (81.0 IU/mL) and fed-batch culture (144.0 IU/mL) as compared to the other reported *T. reesei* strains in the literature grown in batch or fed-batch experiments on cellulose or lactose. This superiority of SEU-7 over RUT-C30 improves markedly the saccharification ability of SEU-7 on pretreated corn stover. The overexpression of gene BGL1 was found either at the mRNA or at the protein level in the mutant strains with increased cellulase production in comparison with RUT-C30, but only SEU-7 displayed much higher expression of gene BGL1 on lactose than on cellulose. Two copies of gene BGL1 were inserted into the chromosome of *T. reesei* SEU-7 between KI911141.1:347357 and KI911141.1:347979, replacing the original 623-bp fragment that is not within any genes’ coding region. The qRT-PCR analysis revealed that the mRNA levels of both cellulase and hemicellulase were upregulated significantly in SEU-7, together with the MFS transporter CRT1 and the XYR1 nuclear importer KAP8.

**Conclusions:**

Recombinant *T. reesei* SEU-7 displays hyper-production of both cellulase and hemicellulase on lactose with the highest FPase activity and pNPGase activity for *T. reesei*, enabling highly efficient saccharification of pretreated biomass. For the first time, the induction efficiency for cellulase production by lactose in *T. reesei* was reported to be higher than that by cellulose. This outperformance of *T. reesei* SEU-7, which is strain-specific, is attributed to both the overexpression of gene BGL and the collateral mutation. Moreover, the increased transcription levels of cellulase genes, the related transcription factors, and the MFS transporter CRT1 contribute to the outstanding cellulase production of SEU-7. Our research advances strain improvement to enhance the induction efficiency of soluble carbon sources to produce cost-effective cellulase and hemicellulase in industry.

**Electronic supplementary material:**

The online version of this article (doi:10.1186/s13068-017-0915-9) contains supplementary material, which is available to authorized users.

## Background

Although the insoluble cellulosic materials are considered as the most effective natural inducers for cellulase production by microorganisms in terms of both enzyme yield and productivity, their insolubility causes many problems and presents a major drawback that is partly responsible for the high cost of the cellulase production. First, the insolubility of cellulose leads to difficult and complex fermentation operations, including sterilization, cell biomass measurement, mixing and aeration of the fermentation broth [1], continuous feeding/sampling, and subsequent enzyme purification [2]. Second, cellulase gets absorbed to the solid cellulose surface, leading to enzyme loss. Third, cell growth and cellulase production are dependent on the cellulose hydrolysis, making the whole process time-consuming. Lastly, cellulase production induced by cellulose is very energy-intensive because of vigorous mixing and aeration of the viscous non-Newtonian fermentation broth [1]. These problems could be overcome if soluble inducers are used.

Previous studies showed that some soluble carbon sources alone can also induce the cellulase production, like sophorose [3, 4], cellobiose [5], galactose, l-sorbose [6], and lactose [7]. Sophorose is a strong inducer of cellulase, but it is very costly, and thus is not a practical carbon source for cellulase production [3]. The inducing effects of other soluble carbon sources are inferior to cellulose, partly due to central carbon catabolite repression (CCR).

Lactose, accumulated as the byproduct of cheese production, is inexpensive and economically feasible. Thus, it has become a major industrial inducer for (hemi)cellulolytic enzyme fermentation by *T. reesei,* though it is still less efficient in cellulase induction than cellulose owing to its slower induction rate and lower yield [5]. To improve the induction efficiency of lactose, several studies on strain improvement and fermentation process optimization have been carried out. For example, *T. reesei* mutant C-5 derived from QM9414 by mutagenesis with NTG can produce cellulase with 2.84 IU/mL FPase activity (Filter paper activity) on lactose [7], while overexpression of XYR1 under a copper responsive promoter in the ΔXYR1 strain derived from *T. reesei* QM9414 merely resulted in super low pNPCase (p-nitrophenyl-β-cellobioside) and pNPGase (*p*-*n*-nitrophenyl-β-glucosidase) activities on lactose [8]. The supplement of other carbon sources such as glucose [9] and cellulose + lactobionic acid [10], and different fermentation strategies like fed-batch culture [11] and continuous culture [9], have been utilized to help enhance the induction efficiency of lactose. These strategies are sometimes explored in combination to achieve a better induction effect. Despite the efforts shown above, the lactose induction efficiency is still low and not satisfying for cellulase industrial application.

In this research, *T. reesei* mutant SEU-7, derived from *T. reesei* RUT-C30 using insertional mutagenesis with AMT, displayed remarkably enhanced cellulase and hemicellulase production on both lactose and cellulose. For the first time, the cellulase production on lactose was reported to be superior to cellulose in *T. reesei*. The cellulase production of SEU-7 on lactose was further improved by optimization of lactose concentration, utilization of fed-batch culture, and supplement of low-concentration glucose, resulting in record FPase and pNPGase activities by *T. reesei*. Accordingly, improvement of the saccharification ability of SEU-7 cellulase on pretreated corn stover was observed. The role of the overexpression of gene BGL1 and the collateral mutation on the outperformance of SEU-7 were explored by profiling all other recombinants and determining the inserted copy numbers and the insertion sites of gene BGL1. The molecular mechanism for the outstanding performance of SEU-7 was further investigated by strain characterization and transcription analysis of the relevant genes.

## Methods

### Strains, plasmids, and culture media

Plasmid construction and propagation were carried out in *Escherichia coli* DH5α. *A. tumefaciens* AGL-1 was utilized as a T-DNA donor for the transformation of *T. reesei* RUT-C30 (CICC 13052) by *A. tumefaciens*-mediated transformation (AMT) [12]. *E. coli* DH5α and *A. tumefaciens* AGL-1 were grown in LB medium with 220 rpm at 37 and 28 °C, respectively. *T. reesei* was cultivated on potato dextrose agar (PDA) plate for conidia culture and in *Trichoderma* minimal media (TMM) [13] with 2% (w/t) cellulose or other carbon sources as indicated in the context for cellulase production at 28 °C with 200 rpm. The TMM medium was composed of the following (all concentrations in g/L unless otherwise noted): urea, 1.00; (NH_4_)_2_SO_4_, 4.00; KH_2_PO_4_, 6.59; FeSO_4_·7H_2_O, 0.005; MnSO_4_·H_2_O, 0.0016; ZnSO_4_·7H_2_O, 0.0014; CoCl_2_·6H_2_O, 0.002; MgSO_4_, 0.60; CaCl_2_, 0.60; Tween-80, 0.0186% (v/v); Tryptone, 0.75; Yeast extract, 0.25, Maleic acid, 11.6. The pH of TMM was adjusted to 6 by NaOH. Plasmid pDht/sk was a gift from Professor Zhihua Zhou from Key Laboratory of Synthetic Biology, Shanghai [14]. All chemicals used in this study were purchased from Sigma-Aldrich (St. Louis, MO, USA).

### Construction of recombinant *T. reesei* strains

By using HiScript 1st Strand cDNA Synthesis Kit (Vazyme, China), the first-strand cDNA was synthesized from total RNA of *T. reesei* RUT-C30 that was extracted with the RNA extraction Kit (Omega Bio-Tek, USA). BGL1 gene was amplified from *T. reesei* RUT-C30 cDNA with primers listed in Additional file 1: Table S1, and cloned into the backbone plasmid pDht/sk at *Xba*I using the ClonExpress II One Step Cloning Kit (Vazyme, China), generating plasmid pBGL-his (Additional file 1: Figure S1). Plasmid pBGL-his was transformed into *T. reesei* RUT-C30 by the AMT method [12]. After transferring the putative transformants on PDA medium containing hygromycin B successively for five generations, single spore colonies were isolated and five recombinant *T. reesei* strains were obtained: SEU-2, SEU-5, SEU-6, SEU-7, and SEU-8, in which the insertion of BGL1 gene was confirmed by PCR and sequencing.

### Cellulase production

10^7^/mL conidia produced by *T. reesei* SEU-7 or RUT-C30 grown on PDA plates for 7 days at 28 °C, which were counted by a Petroff-Hausser cell counter (American Optical, USA), were inoculated into 50-mL Erlenmeyer flasks containing 10 mL Sabouraud dextrose broth (SDB) and incubated for 48 h with 200 rpm at 28 °C. Pre-grown mycelia were inoculated with an inoculation ratio of 10% (v/v) into 250-mL flasks containing 50 mL TMM media (pH 6) with microcrystalline cellulose (Sinopharm Group Co. LTD), lactose, galactose, sucrose, cellobiose, glycerol, or glucose of different concentrations as shown in the context, and then incubated at 28 °C with 200 rpm. The fed-batch culture was carried out in the same way as the above batch culture except that 1.5 g autoclaved lactose powder, whose final concentration was 3%, was added at 120 h together with 5 mL 10 × TMM medium in one pulse. The volume of broth was 42 mL at the end of fermentation. When testing the influence of glucose supplementation on enzyme activities and protein concentration, 0.05–5% glucose was added into the TMM medium with 3% lactose or 2% cellulose as the inducer. Samples at different time points were taken for enzyme assay. Specifically, 1 mL culture was taken and centrifuged at 8000 rpm for 15 min at 4 °C to remove *T. reesei* cells and other solid materials, and the supernatant was stored at − 80 °C for further study. Each experiment was performed in three biological replicates.

### Enzyme assay

The collected culture supernatant was diluted properly for enzyme assay. Enzyme activities of cellulase were measured by the standard protocols established in our previous study [15]. β-xylosidase activity was measured by using 4-nitrophenyl-β-d-xylopyranoside as a substrate dissolved in a 50 mM sodium citrate buffer, pH 5.0. The enzyme assay was performed at 50 °C as previously described [16]. Xylanase activity was determined with 1% beech xylan in 50 mM sodium acetate buffer as substrate [17]. 50 μL diluted culture supernatants were mixed with 50 μL substrate and reacted at 50 °C for 10 min. The reaction was stopped by adding 100 μL alkaline 3,5-dinitrosalicylic (DNS) and heating for 5 min at 100 °C. Then the absorbance at 540 nm was detected. Protein concentration was determined using BCA Protein Assay Kit (Sangon Biotech, China). All enzyme activities were presented as activities using international units (IU) per mL supernatant. One IU was defined as the amount of enzyme required to liberate 1 μmol of product per minute under the standard assay conditions. All experiments were performed in triplicate. The enzyme activities of the fed-batch experiment are calculated with the corrected broth volume to eliminate the concentration effect caused by evaporation.

### Analysis method

Glucose was measured following the instructions of Glucose Detection Kit (Shanghai Rongsheng Biotech, China). For biomass measurement, 3 mL solid biomass was washed with tap water three times, and dried at 50 °C to constant weight [11]. DNA was determined as described in the literature [18]. SDS-PAGE analysis was performed on 10% Tris–HCl polyacrylamide gels using 10 μL cell culture of different *T. reesei* strains from day 7.

### Saccharification of corn stover pretreated with EDA or alkali

Corn stover was harvested in suburb of Tianjin (China), air-dried, milled, and passed through a 2-mm sieve before pretreatment. The moisture of the milled corn stover was 4%. The milled corn stover was pretreated by ethylenediamine (PCS-EDA) in a vacuum drying oven as previously described [19, 20], which was termed as PCS-EDA. Corn stover containing 37.2% glucan and 19.2% xylan harvested in suburb of Jiangsu was air-dried, milled, and passed a 4-mm sieve with the moisture of 11% before pretreatment and treated by 2% dilute alkaline in a 1-L conical flask at 121 °C, 20 min [21], which was termed as PCS-alkali. Pretreated biomasses were then washed using water for several times until neutral pH. The washed biomasses were dried at 70 °C in an oven and stored at 4 °C before use.

The compositions of the pretreated corn stover (PCS) were determined following the Laboratory Analytical Procedure (LAP) of the National Renewable Energy Laboratory (NREL). We found that the PCS-EDA has 27.9% glucan, 9.9% xylan, and 16.6% acid-insoluble lignin in dry matter and the PCS-alkali 37.9% glucan, 19.2% xylan, and 16.7% acid-insoluble lignin. The other components in PCSs, such as soluble lignin, proteins, lipids, salts, were not analyzed here, leading to the PCS mass balances are well below 100%.

The supernatant collected at 168 h in batch culture and crude enzymes at 192 h in the fed-batch culture were used for saccharification. 10% (w/v) pretreated biomass in 1.5 mL buffer (50 mM sodium citrate buffer at pH 5.0 with 1 mM sodium azide to prevent microbial contamination) and different amount of crude enzyme were mixed and incubated at 50 °C with 400 rpm for 72 h. The reducing sugar level in the supernatant was determined by the DNS method. The experiments were performed in three biological repetitions.

### Copy number assay

The copy numbers of the integrated gene BGL1 in the transformant strains SEU-2, 5, 6, 7, and 8 were determined by qPCR using extracted genomic DNA as the template and primers (Additional file 1: Table S2) on the basis of the reported method [22, 23]. Genomic DNAs of *T. reesei* strains were extracted individually with the E-Z 96 Fungal DNA Kits (Omega Bio-tek, Germany). Genes CEL7A, PGK1, and SAR1 were used to represent the single-copy genes, which is confirmed by nucleotide blasting against *T. reesei* genome sequence using the *T. reesei* genome database v2.0 (https://www.ncbi.nlm.nih.gov/genome/323?genome_assembly_id=49799). The qPCR was performed as follows: 95 °C for 30 s, 1 cycle of 95 °C for 15 s, 60 °C for 34 s, 40 cycles and 95 °C for 15 s, 60 °C for 60 s, 95 °C for 15 s 1 cycle. Three biological replicates were performed with three technical replicates for each biological replicate.

### Whole genome resequencing

To identify the insert sites of gene BGL1 in the chromosome of *T. reesei* SEU-7, the genomes of SEU-7 and RUT-C30 were re-sequenced with Illumina HiSeq instrument according to manufacturer’s instructions (Illumina, San Diego, CA, USA). The NGS library was constructed following the manufacturer’s protocol (Illumina TruSeq DNA Nano Library Prep Kit). Then libraries with different indices were multiplexed and loaded on an Illumina HiSeq instrument according to manufacturer’s instructions (Illumina, San Diego, CA, USA). Sequencing was carried out using a 2 × 150 paired-end (PE) configuration; image analysis and base calling were conducted by the HiSeq Control Software (HCS) + OLB + GAPipeline-1.6 (Illumina) on the HiSeq instrument. The library construction and sequences were processed and analyzed by GENEWIZ (Su Zhou, China) using *T. reesei* RUT-C30 genome database (https://www.ncbi.nlm.nih.gov/genome/323?genome_assembly_id=49799). The corresponding fastq files of NGS for SEU-7 was deposited into NCBI SRA database with the NCBI Accession Number: SRR5931428.

### qRT-PCR analysis

Reverse transcription (RT) was performed using the AceQ qPCR SYBR Green Master Mix (Takara, China) with total RNA that was prepared as described above from fresh mycelia of *T. reesei* SEU-7 and RUT-C30 cultivated under different conditions. The RNA concentration was determined at 260 nm using a NanoDrop ND-2000 (Thermo Fisher Scientific, USA). qRT-PCR was performed on the ABI StepOne instrument Plus (ABI, Germany) with software Version 2.3 (ABI, Germany). The primers for qRT-PCR are shown in Additional file 1: Table S3. At least three biological replicates were performed with three technical replicates for each biological replicate. The expression of PGK1 was chosen as the reference gene for data normalization [24].

## Results

### Recombinant *T. reesei* strain SEU-7 exhibits superior cellulase production ability for both cellulose and lactose to RUT-C30

The random insertion of a certain gene into a fungus’s chromosome for gene overexpression by the T-DNA-based mutagenesis usually interferes or disrupts other genes’ expression, leading to varied performance of different recombinant strains [5, 15, 24]. This phenomenon can be utilized as a powerful mutagenesis method in addition to the traditional ones using nitrosoguanidine (NTG) or UV irradiation. Here, endogenous gene β-glucosidase (BGL1, containing a linker and a C-terminal 6-histidine tag) was overexpressed in *T. reesei* under a modified CBH1 promoter [14] through the random insertion by AMT using the plasmid pBGL-his (Additional file 1: Figure S1A) to search for mutant strains with improved performance for cellulase production. Five transformants SEU-2, 5, 6, 7, and 8 were obtained with the cellulase production on cellulose assayed on day 5 (Additional file 1: Figure S1B). The pNPGase and CMCase (carboxymethyl cellulase) activities of all the tested recombinants were higher than the parental strain RUT-C30. However, the pNPCase activity of strains SEU-2 and SEU-5 was decreased, leading to the reduced FPase activity in these two strains. Although the strains SEU-6 and SEU-8 shared a similar pNPCase activity with RUT-C30, their FPase activity was lower than RUT-C30. Only the strain SEU-7 displayed increased pNPCase activity and FPase activity. Obviously, the strain SEU-7 with the highest cellulase activities outperforms other recombinant strains as well as the parent strain RUT-C30, and was therefore selected for further study.

**Fig. 1:**
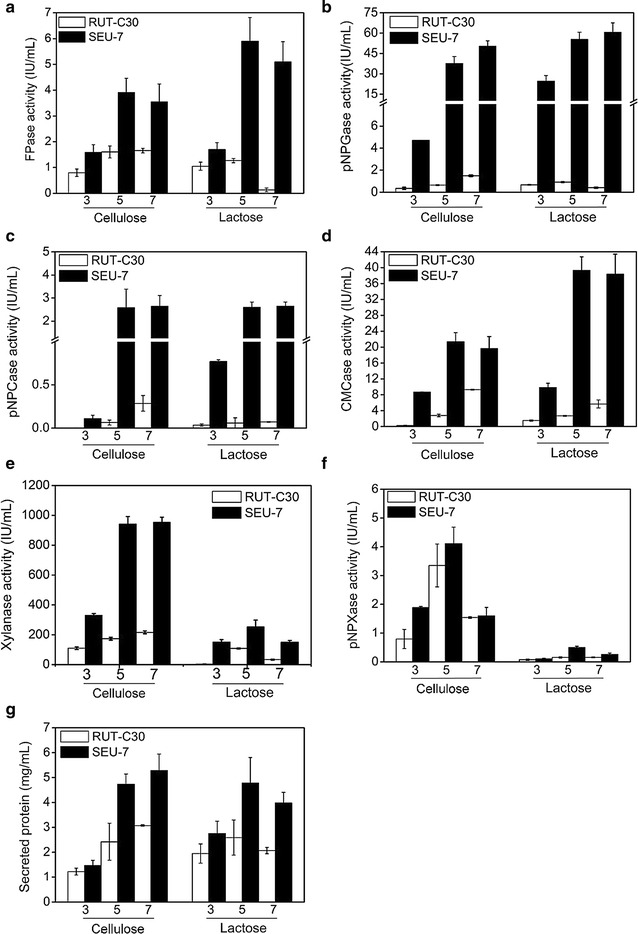
Cellulolytic enzyme activities in the culture supernatant of *T. reesei* SEU-7 and RUT-C30 grown on 2% cellulose or 2% lactose were assayed on day 3, day 5, and day 7, including the activities of FPase (the filter paper activity) (**a**), pNPGase (the BGL activity) (**b**), pNPCase (the CBH activity) (**c**), CMCase (the CMC activity) (**d**), xylanase (**e**) and pNPXase (the β-xylosidase activity) (**f**), and the protein concentration (**g**). The error bars indicate the standard deviations of three replicates

Using cellulose as the sole carbon source, the FPase activity of the strain SEU-7 was 2.0-, 2.4-, and 2.2-fold that of RUT-C30 on days 3, 5, and 7, respectively (Fig. 1a). SEU-7 displayed 50 IU/mL pNPGase activity on day 7 (Fig. 1b), which was 34-fold that of RUT-C30. The pNPCase activity was increased by eightfold in SEU-7 on day 7 (Fig. 1c). The maximal CMCase activity of SEU-7 (21.3 IU/mL) was observed on day 5 and was 7.6-fold higher than that of RUT-C30 (2.8 IU/mL on day 7) (Fig. 1d). In agreement with the noticeable increment of cellulase activities, 42% more protein production was detected in the supernatant of SEU-7 cell culture (Fig. 1g). All together, SEU-7 outperforms the parent strain RUT-C30 by displaying markedly increment of both cellulase activities and secreted protein amount.

When grown on lactose, the maximal activities of FPase, pNPGase, pNPCase, and CMCase of SEU-7 were measured to be 5.9, 60.6, 2.6, and 39.0 IU/mL, respectively, which were 4.5-, 152.0-, 43.0- and 14.4-fold those of RUT-C30 on lactose, and were 3.7-, 40.6-, 43.0- and 14.0-fold those of RUT-C30 on cellulose (Fig. 1). As compared to those of SEU-7 grown on cellulose, the maximal activities of FPase, pNPGase, and CMCase of SEU-7 on lactose were improved by 51, 22, and 86%, respectively, while the pNPCase activity and the protein production were comparable. Obviously, the cellulase production of strain SEU-7 on 2% lactose is even better than that on 2% cellulose, which is in contrast to that result that the cellulase activities of RUT-C30 were compromised substantially on lactose as compared to cellulose (Fig. 1). The extracellular protein concentration of SEU-7 on lactose was also found to increase significantly 0.9-fold in comparison to RUT-C30 on lactose, demonstrating that the protein secretion ability of SEU-7 was improved. In summary, the induction efficiency of lactose was increased significantly in strain SEU-7, resulting in that the cellulase production of SEU-7 on lactose is higher than that of the industrial strain RUT-C30 on both cellulose and lactose.

SEU-7 exhibits better performance than RUT-C30 when grown on other soluble carbon sources including cellobiose, sucrose, galactose, glycerol, and glucose for cellulase production, but these carbon sources are not better cellulase inducers than cellulose or lactose for both strains (Additional file 1: Figure S2).

In addition, enhanced hemicellulase activity was also found in strain SEU-7 grown on either lactose or cellulose. The maximal xylanase activity of strain SEU-7 was measured to be 953 IU/mL on cellulose on day 7 and 253 IU/mL on lactose on day 5, which were 4.4- and 2.3-fold of those of RUT-C30, respectively (Fig. 1e). The final pNPXase (*p*-nitrophenyl-β-d-xylopyranoside) activity of SEU-7 was comparable to RUT-C30, although SEU-7 exhibited an increased pNPXase activity in the early phase day 3 (Fig. 1f).

### The cellulase production of SEU-7 on lactose was further improved by lactose concentration optimization and fed-batch culture

**Fig. 2:**
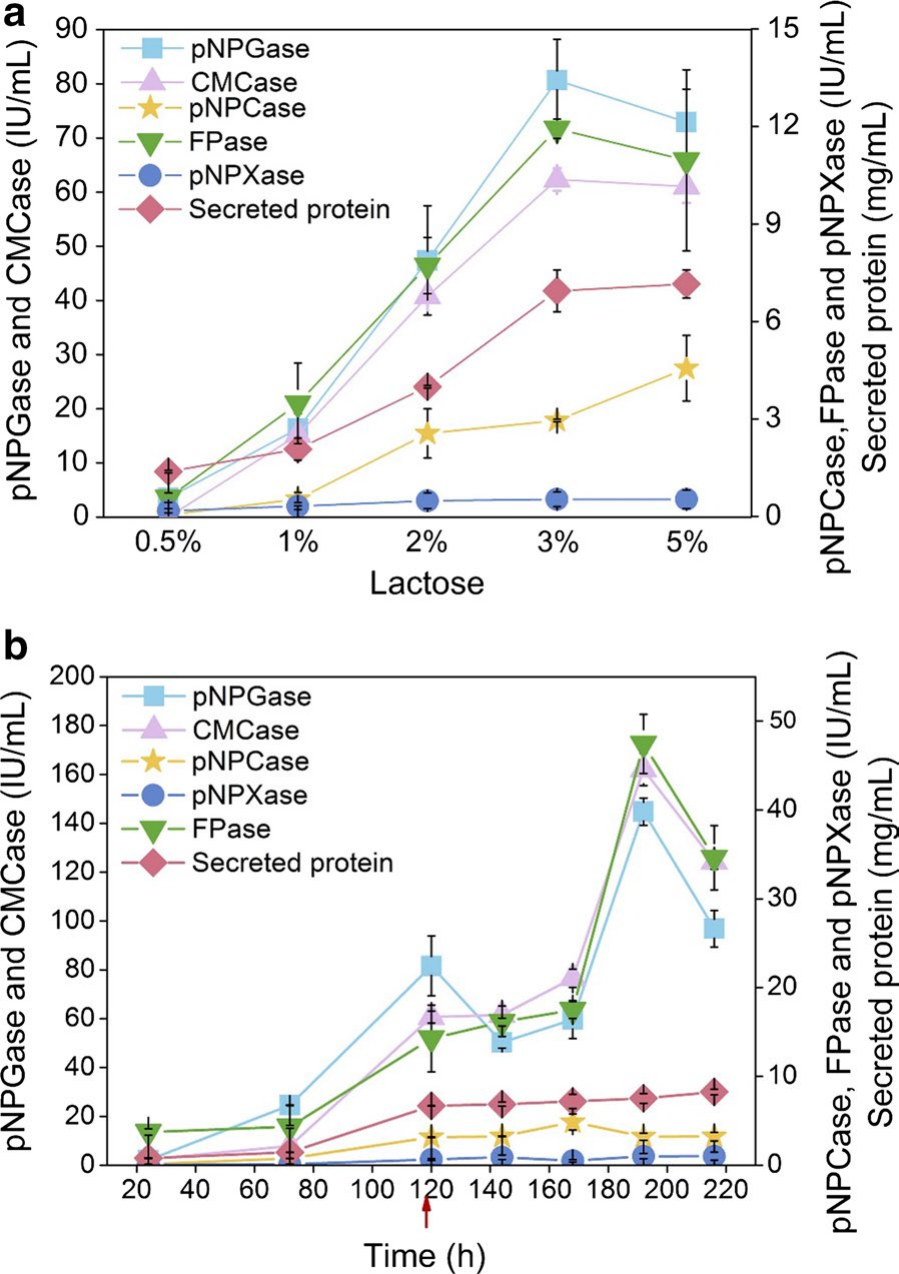
Cellulolytic enzyme activities of *T. reesei* SEU-7 and RUT-C30 grown on 0.5–5% lactose for 5 days (**a**) and cellulolytic enzyme activities of *T. reesei* SEU-7 in the fed-batch culture with 3% lactose (**b**). The error bars present the standard deviations of three biological replicates. The red arrow indicated the feeding time point

It is reported that the cellulase production of *T. reesei* is dependent on the lactose content in the fermentation medium [7, 25, 26]. Therefore, the effect of lactose concentration on the cellulase production of strain SEU-7 was investigated (Fig. 2a). All the cellulase activities were increased gradually along with the increase of lactose concentration from 0.5 to 3%, and reached a plateau phase beyond 3% lactose, showing 3% lactose has the best induction effect for cellulase production in SEU-7. The FPase, pNPGase, pNPCase, CMCase, and pNPXase activities of SEU-7 induced by 3% lactose were 13.0, 81.0, 3.0, 62.0, and 0.5 IU/mL, respectively (Fig. 2a). When fed-batch culture with 3% lactose as the sole carbon source was explored, the maximum activities of FPase, pNPGase, and CMCase were 47.0, 140.0, and 164.0 IU/mL, respectively, at 192 h, while the maximal activities of pNPCase and pNPXase were 4.8 IU/mL at 168 h and 0.9 IU/mL at 144 h, respectively (Fig. 2c). The productivity and the yield of cellulase production were calculated using the FPase activity. When calculating the yield, it is assumed that lactose in the culture was metabolized completely as the lactose concentration was not measured. The FPase productivity was 0.10 and 0.24 IU/mL/h for the batch experiment and fed-batch experiment, repetitively, and the FPase yield individually 397 and 783 IU/g. The FPase productivity and yield was up 1.4- and 1.0-fold, respectively, by using fed-batch. The FPase productivity was higher than that of other previous studies with 0.2 [27] and 0.038 IU/mL/h [9] reported using lactose as inducer. Clearly, the cellulase production of SEU-7 on lactose was significantly enhanced by lactose concentration optimization and fed-batch culture.

### The cellulase production in strain SEU-7 was enhanced by the supplement of low-concentration glucose

**Fig. 3:**
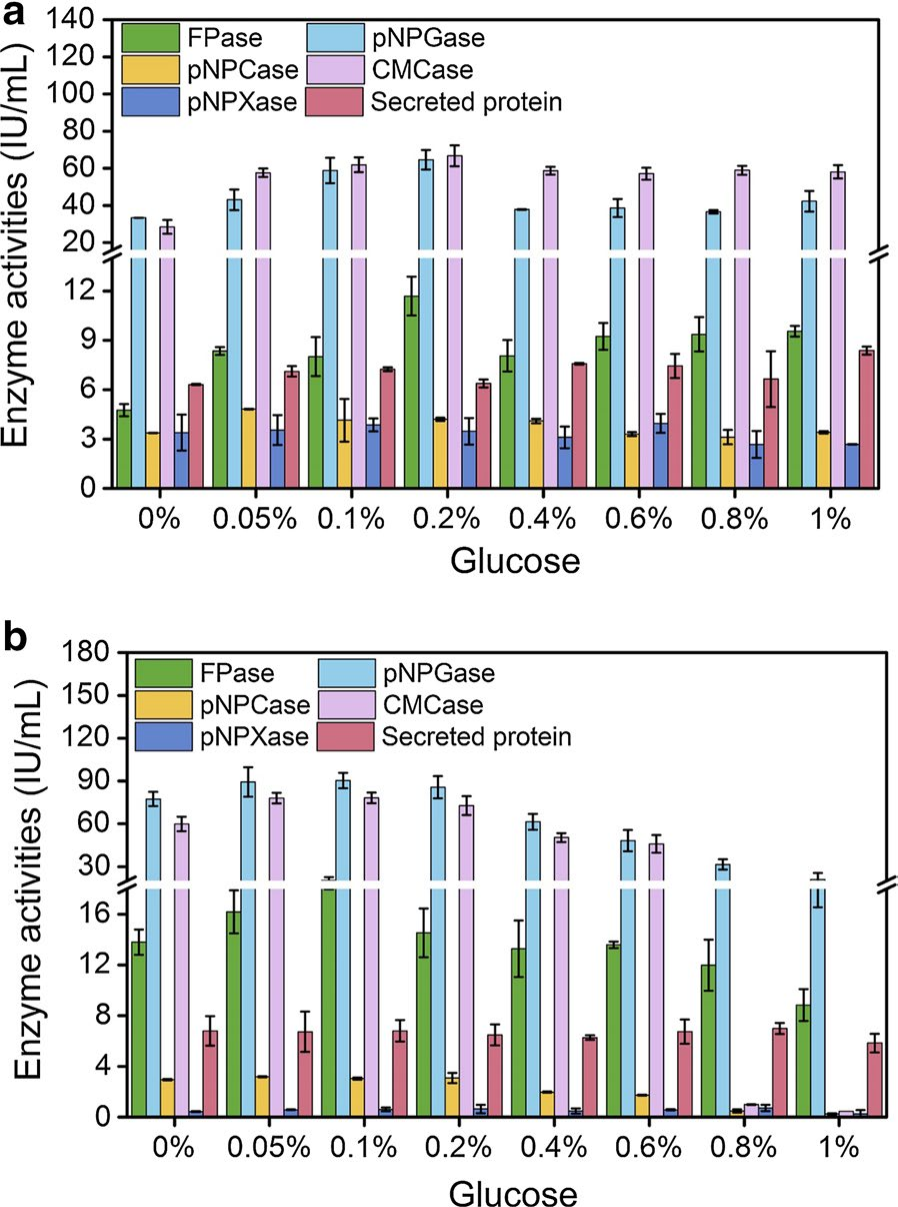
Effect of 0–1% glucose on cellulase production of strain SEU-7 grown on 3% lactose (**a**) or 2% cellulose (**b**). Cellulolytic enzyme activities were all measured on day 5. The error bars present the standard deviations of three biological tests

As the sole carbon source in the medium, lactose serves as both an inducer for cellulase production and the carbon source for mycelium growth. The sharing of lactose for growth might limit the cellulase production. Given that strain SEU-7 displays a better resistance to CCR than RUT-C30 (Additional file 1: Figure S3), we envision that the supplement of glucose with low concentration for cell growth will not cause CCR, and will improve the cellulase production of SEU-7. Therefore, glucose in the range of 0–1% was added into the TMM medium containing 3% lactose and the cellulase activities were measured (Fig. 3a). The FPase activity kept rising from 13.0 to 20.0 IU/mL when the glucose concentration was increased from 0 to 0.1%, beyond which the FPase activity was decreased gradually. The pNPGase activity and the CMCase activity followed a similar trend as the FPase activity along with the increment of glucose concentration, peaking at 0.1% glucose. The maximal activities of pNPGase and CMCase were measured to be 90.0 and 78.0 IU/mL, respectively, which were improved by about 14.2 and 23.4%, respectively, when compared to those in the absence of glucose. The pNPCase activity stayed stable at 0–0.2% glucose, but dropped sharply when the glucose concentration used was > 0.2%. The protein concentration held steady until the glucose concentration was higher than 0.8% where it started to decline. In summary, the supplement of 0.1% glucose with 3% lactose has improved most cellulase production of SEU-7.

The benefit of the supplement of glucose was also observed with 2% cellulose as the carbon source. The maximal activities of FPase, pNPGase, and CMCase were found to be 12.0, 65.0, and 67.0 IU/mL, respectively, in the presence of 0.2% glucose (Fig. 3b). Furthermore, the pNPCase activity, pNPXase activity, and protein concentration were not changed as the glucose concentration varied. Apparently, the cellulase production of SEU-7 on lactose was more affected than on cellulose, when the mixed glucose concentration was increased from 0 to 1%, indicating that SEU-7 on lactose is more vulnerable to CCR than cellulose.

### Characterization of *T. reesei* SEU-7 on lactose and cellulose

**Fig. 4:**
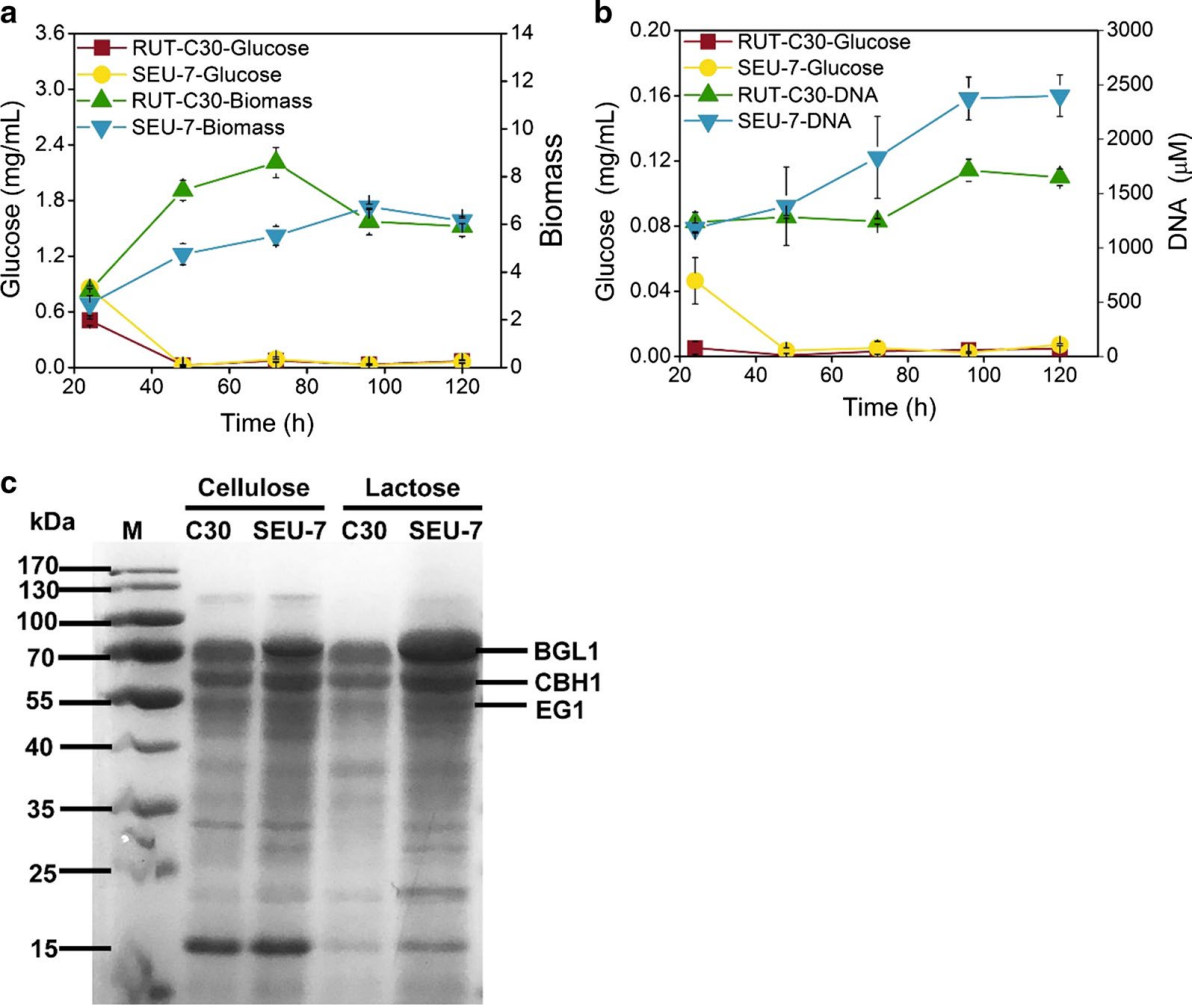
Growth and glucose concentration in the culture supernatant of *T. reesei* RUT-C30 and SEU-7 on 3% lactose (**a**) or 2% cellulose (**b**). SDS-PAGE analysis of secretome of RUT-C30 and SEU-7 grown on 3% lactose or 2% cellulose for 7 days (**c**)

Although the strain SEU-7 displayed markedly improved cellulase activities on both lactose and cellulose than RUT-C30, its growth on lactose was noticeably slower than the strain RUT-C30 with 63% of the RUT-C30 biomass at 72 h but reached the same biomass as RUT-C30 at 96 h due to the steeply drop of the RUT-C30 biomass after 72 h (Fig. 4a). On cellulose, however, SEU-7 was grown better than RUT-C30 after 48 h with 1.5-fold more DNA content detected at 120 h (Fig. 4b). Due to the interference of insoluble cellulose to the measurement of dry *T. reesei* biomass, measurement of DNA content was used as an alternative method for biomass measurement.

69% more glucose was detected in the supernatant of SEU-7 than RUT-C30 on lactose at 24 h, after which the glucose concentration of both strains fell sharply to the identical concentration of 0.2 mg/mL at 48 h. It remained unaltered afterwards (Fig. 4a). When using cellulose as the carbon source, the strain SEU-7 had a very low glucose concentration (0.04 mg/mL) in its supernatant of 24 h, eightfold that of RUT-C30, which was followed by a sharply decline to 0.003 mg/mL at 48 h (Fig. 4b) and stayed unchanged in the rest of the fermentation process. In contrast, there was no obvious change of glucose concentration (~ 0.005 mg/mL) in the culture supernatant of RUT-C30 throughout the whole process of cellulase production (Fig. 4b). That more glucose was detected in the supernatant of SEU-7 culture on both lactose and cellulose might partly be attributed to significant enhancement of BGL activity found in SEU-7 on both carbon sources (Fig. 1b).

For both SEU-7 and RUT-C30, the glucose concentration is higher on lactose than on cellulose. This higher glucose concentration might cause more serious CCR, and partly contributes to the less efficient cellulase production of RUT-C30 on lactose. This did not happen to SEU-7 because of its relieved carbon catabolite depression: SEU-7 exhibited higher cellulase activities on glucose than RUT-C30 (Additional file 1: Figure S3).

The secretomes of *T. reesei* SEU-7 cultured in TMM with lactose or cellulose on day 7 were profiled by SDS-PAGE with equal volume of the culture supernatant (Fig. 4c). Obviously, SEU-7 secreted more cellulase than RUT-C30 into the culture medium, which is consistent with the observed increases of both cellulase activities (Fig. 1a) and protein concentration (Fig. 1g). It seems that SEU-7 possesses a better protein secretion ability than RUT-C30 when cultured on either lactose or cellulose.

### The biomass saccharification ability of cellulase produced by strain SEU-7 was enhanced significantly

**Fig. 5:**
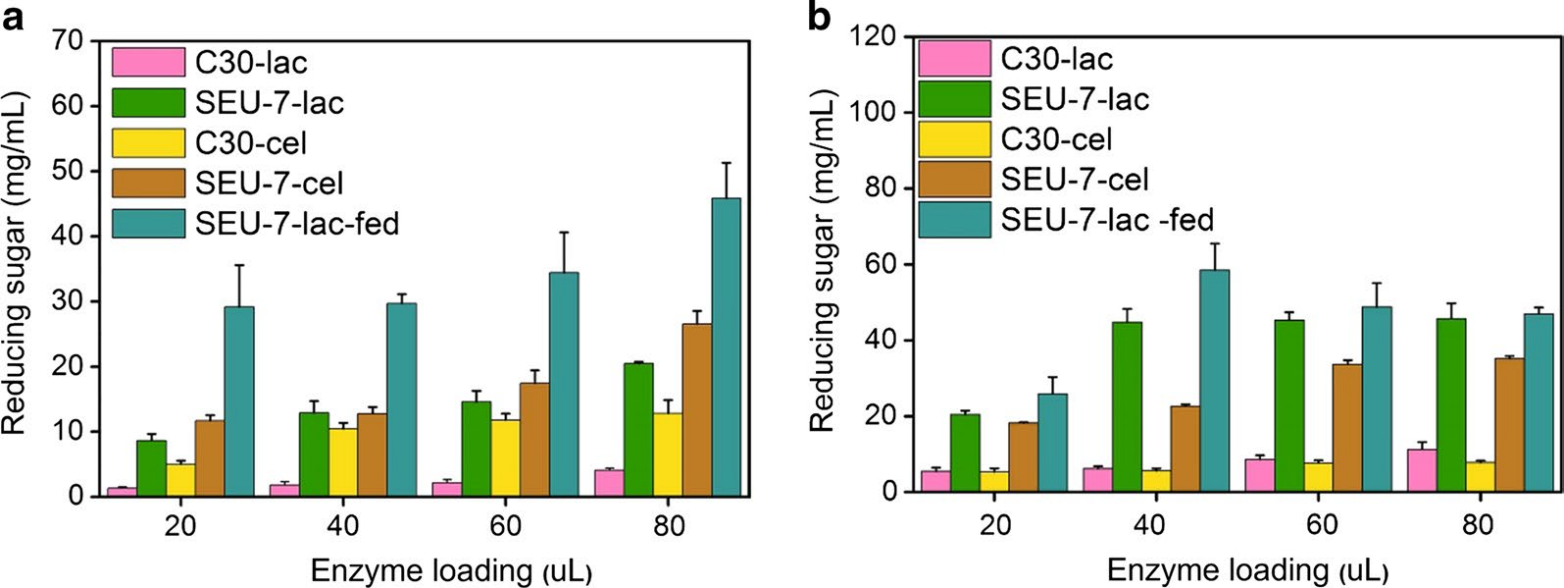
Saccharification of PCS with enzyme loading from 20 to 80 μL by *T. reesei* RUT-C30 and SEU-7 using the equal culture supernatants of RUT-C30 and SEU-7 on 2% cellulose or 3% lactose, respectively. Saccharification of corn stalk pretreated with EDA (**a**) or alkali (**b**) by crude enzyme solution produced from strain SEU-7 and RUT-C30 grown on lactose or cellulose, which was designated SEU-7-lac, SEU-7-cel, C30-lac, and C30-cel, respectively. The crude enzyme solution prepared from fed-batch culture of SEU-7 on 3% lactose was named as SEU-7-lac-fed. The error bars indicate the standard deviations of three biological replicates

The hydrolysis efficiencies of crude enzyme solutions produced from the strain SEU-7 and RUT-C30 grown on lactose or cellulose, designated SEU-7-lac, SEU-7-cel, C30-lac, and C30-cel, respectively, were evaluated using corn stover pretreated with EDA (PCS-EDA) or alkali (PCS-alkali) as substrates (Fig. 5). On both substrates, both SEU-7-lac and SEU-7-cel produced much more reducing sugar than C30-lac or C30-cel at the same enzyme loading (Fig. 5). The maximal reducing sugar of SEU-7-lac (20.5 mg/mL with 80 μL enzyme loading) on PCS-EDA was five times that of C30-lac (4.1 mg/mL with 80 uL enzyme loading) (Fig. 5a). The maximal reducing sugar of SEU-7-lac (44.8 mg/mL with 40 μL enzyme loading) on PCS-alkali was fourfold that of C30-lac (11.2 mg/mL with 80 μL enzyme loading) (Fig. 5b). The hydrolysis efficiency of SEU-7-lac was a little lower than SEU-7-avi when using PCS-EDA or PCS-alkali as substrate. The hydrolysis efficiency of crude enzyme solution prepared from fed-batch culture of SEU-7 on lactose (SEU-7-lac-fed) was increased by 60 and 30% for PCS-EDA and PCS-alkali, respectively, as compared to SEU-7-lac. These results show that the mutant strain SEU-7 has much better biomass saccharification ability than RUT-C30 towards both EDA-pretreated biomass and alkali-pretreated biomass, enabling the significantly reduced usage amount of cellulase for hydrolysis of cellulose, which would help greatly reduce the cost of enzyme in industrial application.

### The overexpression of gene BGL1 plays a positive role in the cellulase hyper-production of *T. reesei* SEU-7

**Fig. 6:**
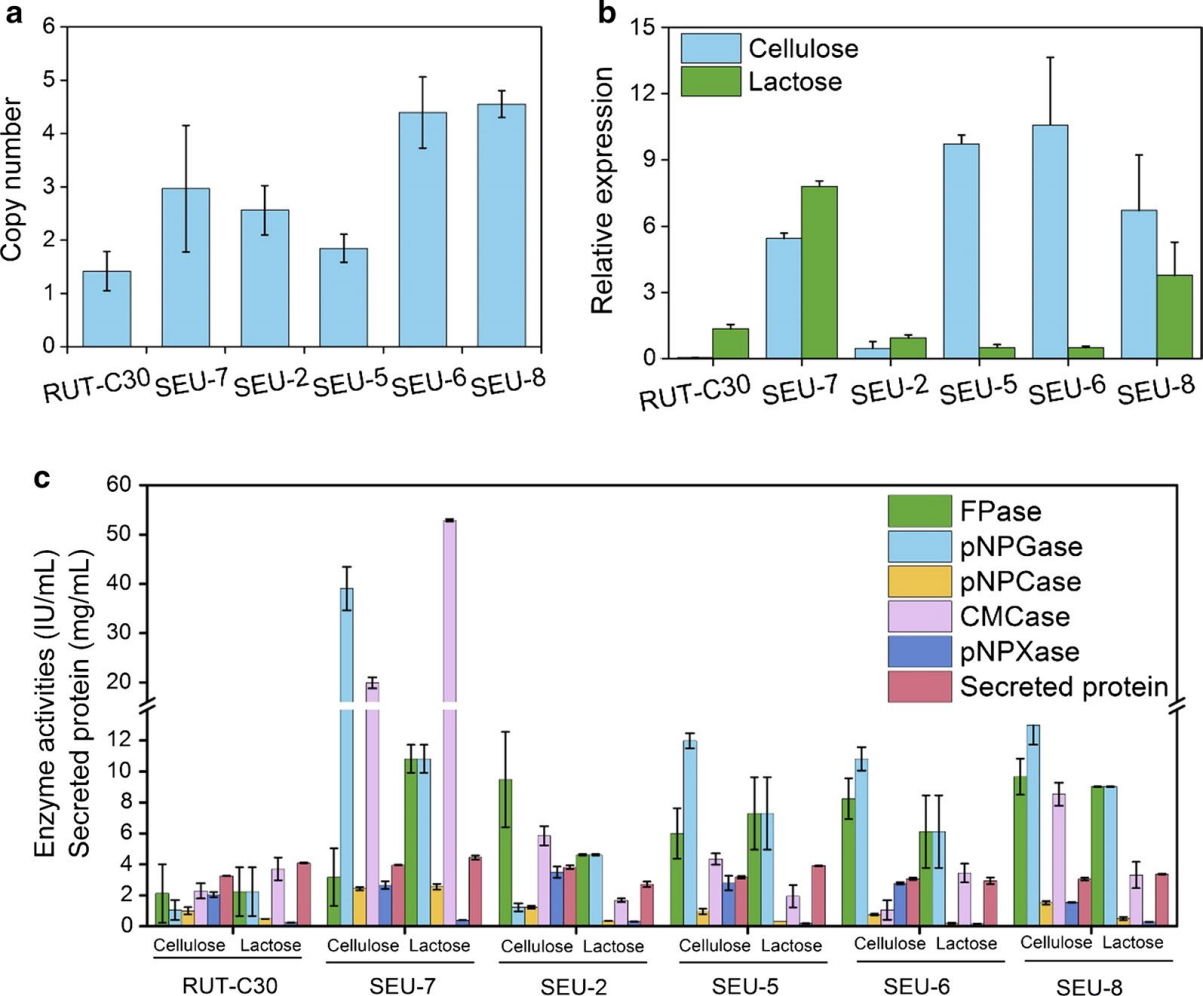
qPCR analysis of the copy numbers (**a**) and the transcript abundance (**b**) of BGL1 gene in *T. reesei* RUT-C30 and the five recombinant *T. reesei* strains: SEU-2, SEU-5, SEU-6, SEU-7, and SEU-8, and the cellulase activities of RUT-C30, SEU-7, SEU-2, SEU-5, SEU-6, and SEU-8 grown on cellulose and lactose for 5 days (**c**)

To see how the overexpression of gene BGL1 impact the excellent performance of *T. reesei* SEU-7, the copy numbers and the mRNA level of gene BGL1, and the cellulase activities for all recombinants obtained in this study were determined. The copy numbers of gene BGL1 inserted were 2, 2, 1, 3, and 3 for strain SEU-7, SEU-2, SEU-5, SEU-6, and SEU-8, respectively, which were measured by qPCR (Fig. 6a). When grown on cellulose or lactose, strain SEU-7 and SEU-8 display a significant increase of BGL production at both the transcriptional level (Fig. 6b) and the protein level (Fig. 6c). Accordingly, SEU-7 and SEU-8 exhibited better cellulase production than RUT-C30 on both cellulose and lactose. A similar trend was observed for strain SEU-5 and SEU-6 on cellulose (Fig. 6c). Although their mRNA level of gene BGL1 was not enhanced on lactose, strain SEU-5 and SEU-6 display a remarkable increase in both BGL protein production and other cellulase production on lactose (Fig. 6c). The seemingly conflicting results between the mRNA expression level and the protein expression level are normal, since many other factors can influence protein expression such as the mRNA lifetime and the protein degradation rate. Conversely, the insertion of one copy of gene BGL1 into SEU-2 genome (Fig. 6a) did not result in the overexpression of gene BGL1 at either the mRNA level (Fig. 6b) or the protein level (Fig. 6c) when it was grown on cellulose or lactose. Meanwhile, SEU-2 exhibits similar cellulase production ability to RUT-C30 on both cellulose and lactose (Fig. 6c). It is worth mentioning that the mRNA level of gene BGL1 in strain SEU-7 on lactose was higher than on cellulose, which might contribute to the higher induction efficiency with lactose than with cellulose in strain SEU-7. On the contrary, it was reduced sharply in the other recombinants except strain SEU-2 on lactose as compared to cellulose. Obviously, the overexpression of gene BGL1 either at the mRNA or at the protein level plays a positive role in the increased cellulase production of *T. reesei*.

### Identification of collateral mutation

The random insertion of gene BGL1 into the chromosome of *T. reesei* SEU-7 is possible to cause collateral mutations and interfere other genes’ expression, and in turn might contribute to the outstanding performance of SEU-7 together with the overexpression of gene BGL1. Hence, it would be impactful if insertional sites of the two inserted gene BGL1 are identified. To this end, the insertional sites were identified by the whole genome resequencing with NGS sequencing. The whole genome resequencing of *T. reesei* SEU-7 generated a total of 17849966 clean reads of 150-bp paired-end reads with mean depth coverage of 69.75 × and Q30 percentage of 88.69%. The obtained clean reads were mapped to the reference genome of RUT-C30, resulting in the high mean coverage of 99.41%. The NGS sequencing result (NCBI Accession Number: SRR5931428) show the two inserted copies of gene BGL1 was at 347357-347979 of *T. reesei* RUT-C30 genome KI911141.1 (https://www.ncbi.nlm.nih.gov/nuccore/572281258/), replacing the native fragment of 623 bp (Additional file 1: Figure S4). However, this replaced fragment was not within the coding region of any genes. The nearest downstream and upstream gene was M419DRAFT_127520 (KI911141.1:345112-346074) and M419DRAFT_6256 (KI911141.1:351617-351930), respectively, which is 1284 and 3639 bp away. Both genes are uncharacterized with unknown function. We tried to determine whether the expression of these two genes are affected by the replacement by measuring their mRNA levels with qRT-PCR, but failed because their transcriptional levels are too low to be detected under our current experimental conditions.

### Transcription levels of (hemi)cellulase genes and regulators in *T. reesei* SEU-7

**Fig. 7:**
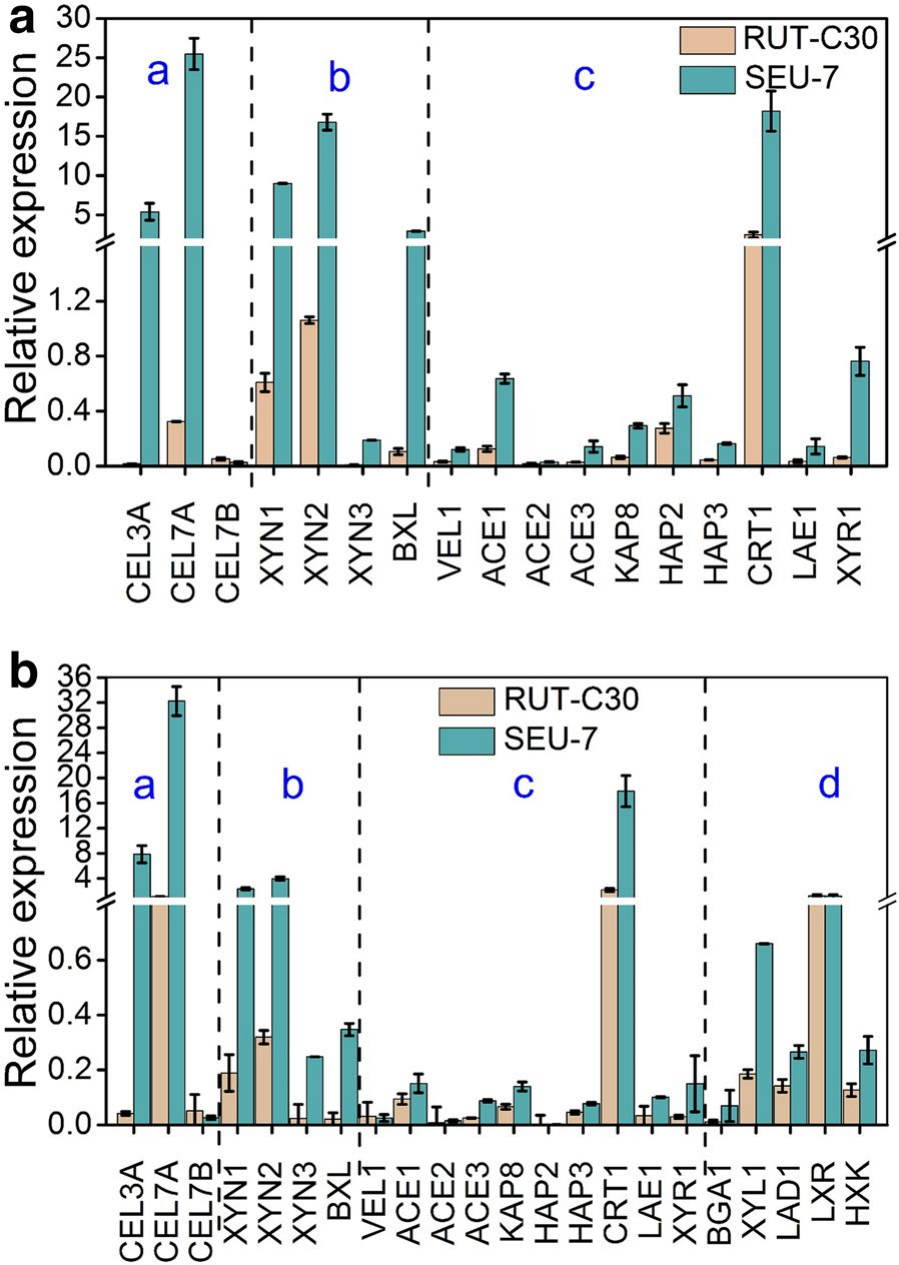
qRT-PCR analysis of the transcript abundance of genes relevant to cellulase and hemicellulase production in *T. reesei* SEU-7 and RUT-C30 grown on 2% cellulose (**a**) or 3% lactose (**b**) for 3 days. a: the main cellulase genes; b: the hemicellulase genes; c: regulatory genes involved in cellulase and hemicellulase production in *T. reesei*; d: genes participated in the lactose metabolism

No matter the carbon source was either lactose or cellulose, the transcriptional levels of (hemi)cellulase genes β-glucosidase BGL1 (CEL3A), cellobiohydrolase CBHI (CEL7A), endoglucanase CMC (CEL7B), β-xylosidase BXL1, and xylanases (XYN1, XYN2, and XYN3) in strain SEU-7 were significantly higher than those of RUT-C30 (Fig. 7), matching well with the markedly enhanced (hemi)cellulase activities and increased (hemi)cellulase production amount found in the supernatant of SEU-7 culture.

Moreover, we analyzed the transcription of genes related to cellulase production, including cellulase transcription activators (XYR1, ACE2, ACE3, and HAP2/3/5), cellulase transcription repressors (ACE1 and CRE1), methyltransferase LAE1, nuclear importer KAP8, major facilitator superfamily (MFS) sugar transporter CRT1, β-glucosidase transcription factor BGLR, and global regulator VEL1(Fig. 7). Genes BGLR, HAP5, and CRE1 were not detected under our experiment conditions. On either lactose or cellulose, the mRNA expressions of both CRT1 and KAP8 were upregulated significantly in SEU-7, while those of LAE1 and VEL1 were unchanged. The transcription level of transcription activator ACE3 was enhanced in SEU-7 on lactose, but stayed constant in SEU-7 on cellulose. In contrast, the other transcription activators (XYR1, ACE2, and HAP2/3) showed constant mRNA levels in both SEU-7 and RUT-C30 on lactose, but displayed upregulated mRNA levels in SEU-7 on cellulose. Unexpectedly, the transcription level of transcription repressor ACE1 was markedly increased in SEU-7 on cellulose, which was not affected at all in SEU-7 on lactose.

The transcription levels of genes involved in lactose metabolism, including genes in both the Leroi pathway (GAL1, GAL7, and GAL10) and the alternative d-galactose pathways (BGA1, XYL1, XDH, HXK, LAD1, and LXR), were assayed in SEU-7 grown on lactose. Genes GAL1, BGA1, XYL1, LAD1, LXR, and HXK were detected. The mRNA levels of XYL1 was elevated remarkably in the strain SEU-7 in comparison to RUT-C30, while other detected genes’ transcription levels remained unchanged, indicating the capacity of these two pathways is abundant and is not a limitation that causes the inefficiency of lactose induction in *T. reesei*.

## Discussion

Recently, several studies have been carried out in improving the induction efficiency of soluble carbon sources, mainly focusing on lactose and glucose that are cheap and abundant. Overexpression of XYR1 under a copper responsive promoter in the ΔXYR1 strain derived from *T. reesei* QM9414 merely resulted in super low pNPCase and pNPGase activities on glucose, lactose, and glycerol [8]. With constitutively overexpression of an artificial transcription activator including the XYR1 effectors, the XYR1 binding domains and the CRE1 binding domain in *T. reesei* RUT-C30, the recombinant strain zxy-2 can produce cellulase with 2.63 IU/mL FPase activity using glucose as a sole carbon source [28]. However, the cellulase production is incomplete with a very low pNPGase activity, leading to the requirement for pNPGase supplement during the saccharification [28]. A sophorose-containing soluble sugar mixture prepared by the transglycosylation reaction catalyzed by β-glucosidase was investigated to obtain an effective synthetic sugar mixture, which can achieve a cellulase titer of 90.3 FPU (filter paper unit)/mL in the fed-batch fermentation of *T. reesei* RUT-C30 on glucose [11]. Nevertheless, this strategy makes the cellulase production process more complex and inconvenient, and introduces extra cost by using the β-glucosidase to carry out the transglycosylation. In addition, the heavy reliance of cellulase production on substrate composition is not flexible and not economic feasible for applications in industry at a large scale. Noticeably, up to now, there are several issues in cellulase production by insoluble carbon sources, including the low productivity, the incomplete compositions of the cellulase production, the extra addition of β-glucosidase for transglycosylation reaction. These issues prevent the economic cellulase production.

Here, *T. reesei* SEU-7 constructed from *T. reesei* RUT-C30 using the T-DNA-based mutagenesis outperforms RUT-C30 in terms of cellulase production on either cellulose or lactose. Most importantly, *T. reesei* SEU-7 is the first *T. reesei* mutant ever reported which cellulase production ability on lactose is better than that on cellulose. The cellulase production of SEU-7 on lactose was further improved by the optimization of lactose concentration, utilization of fed-batch culture, and supplement of low-concentration glucose, resulting in record FPase and pNPGase activities. For SEU-7, the FPase activity of 13.0 IU/mL by batch culture and 47.0 IU/mL by fed-batch culture were 4.6- and 16.5-fold those ever reported (the highest reported FPase activity was 2.84 IU/mL) in *T. reesei* when using lactose as the sole carbon source [8]. To the best of our knowledge, these FPase activities of *T. reesei* SEU-7 when using lactose as the sole carbon source are the highest. Moreover, the pNPGase activity of 81 IU/mL by batch culture and 140.0 IU/mL by fed-batch culture were all higher than the highest BGL activity of 69.7 IU/mL ever reported in *T. reesei* grown on cellulose [22]. This solves the dwindling issue of low β-glucosidase activity in *T. reesei* cellulase that leads to inefficiency in biomass degradation and limits its industrial application [29, 30]. Even better, the pNPCase activity, CMCase activity, and FPase activity of SEU-7 on 3% lactose were all higher than strain T4 on cellulose [22].

Moreover, it is worth mentioning that a complete cellulase production was obtained for SEU-7. Complete enzyme production of SEU-7 enables highly effective hydrolysis of not only EDA-pretreated biomass, but also alkali-pretreated biomass that contains more xylan [11], demonstrating that it allows a broad choice of substrates for saccharification. Meanwhile, improvement of the saccharification ability of SEU-7 compared to RUT-C30 on pretreated corn stover was observed (Fig. 5). These merits make SEU-7 a preferable producer that will work efficiently in the industrial application when cellulose or lactose is utilized as the carbon source for cellulase production.

The benefit of the supplement of low-concentration glucose was observed when using 3% lactose or 2% cellulose as the carbon source, which is probably due to both the relieved CCR and the significantly increased production of β-glucosidase in strain SEU-7. Since strain SEU-7 displays a better resistance to CCR than RUT-C30 (Additional file 1: Figure S3), the supplement of glucose with low concentration did not cause CCR. Instead, the added glucose can serve as the extra carbon source for mycelium growth, saving more lactose or cellulose as the inducer for cellulase production. Moreover, glucose could be transglycosylated by the increased β-glucosidase into sophorose that is a strong inducer for cellulase production [11].

In our opinion, two factors might be responsible for the protein concentration being almost constant, whereas FPase and CMCase activities are multiplied by 2 or 3 after 120 h in the fed-batch experiment (Fig. 2b). Besides cellulase, the secreted protein complex contained non-cellulase proteins like pectinase, mannanase, and the auxiliary proteins which have been continuously found to help improve the hydrolysis of cellulose, such as swollenin (encoded by *swo1*) [31], the Glycoside Hydrolases Family 61 (GH61s) [32], and the cellulase-induced proteins CIP-1 and CIP-2 [33]. No increase in the content of the secreted protein after 120 h does not mean that the amount of cellulase is not increased either. On the other hand, only the three major cellulolytic components, CBH, CMC, and BGL, are not enough for high efficiency of the cellulose hydrolysis, which was demonstrated by the low cellulose hydrolysis yield from the reconstituted mixture consisting of CBH, CMC, and BGL [34]. Low abundant essential enzymes in the supernatant of *T. reesei* culture are also important for the high efficiency of *T. reesei* cellulase. Thus, even if the amount of cellulase is not increased after 120 h, the FPase activity might be improved due to the elevated expression of these low abundant essential enzymes, which is too low to be detected by our protein concentration method.

Genetic engineering has been extensively exploited to increase the BGL activity in *T. reesei*, sometimes as well as the other cellulase compositions, by the overexpression of the native β-glucosidase BGL1 [15, 35] as done in this study or the exotic ones from other fungi including *Neosartorya fischeri* [22], *Penicillium decumbens* [36], *Aspergillus aculeatus* [29, 30], and *Periconia* sp. [37]. Among all these mutant strains, only strain TRB1 has been grown on lactose for cellulase production that is declined in comparison with cellulose [15]. The cellulase production of other recombinants with the overexpression of gene BGL1 in this study like SEU-5, SEU-7, and SEU-8 is more or less compromised on lactose than on cellulase. Moreover, along with the overexpression of BGL1, one or several other cellulase compositions were increased [15, 22, 29, 30, 35, 36] as found in the recombinants SEU-5, SEU-7, and SEU-8 here, but it is rare to get all other cellulase compositions increase as we observed in strain SEU-7. Therefore, the outperformance of SEU-7 on both cellulose and lactose is strain-specific, which might be owing to the fine-tuning of the BGL1 expression to achieve an optimal level, and/or the collateral mutations caused by random insertion.

Two factors might be involved in the role of the BGL1 overexpression playing in the outperformance of strain SEU-7 for cellulase production. The synthetic overexpression of BGL1 both at the mRNA level and at the protein level in strain SEU-7 might play a role in the altered regulatory effect found in strain SEU-7 by transcriptional analysis, which is beneficial for the notable increment of cellulase activities and the overall secreted protein. Besides this, the noticeably improved overexpression of gene BGL1 can increase the sophorose concentration to have more strong inducer for strain SEU-7 to produce cellulase production, for protein BGL1 can catalyze the conversion of glucose and cellobiose to sophorose that is strong inducers for cellulase production [11, 38].

The random insertion of a gene into chromosome for overexpression is possible to interfere other genes’ expression, and in turn affects the performance of *T. reesei* in addition to the overexpression of the gene. As a result, different recombinant strains with the overexpression of the same gene might display varied performance, which was observed in our study. However, this effect in most cases has not been taken into account for the performance of the mutant strains in previous studies [30, 37]. The effect of the insertion position should definitely be considered when dealing with gene modification by random integration. The insertion of two copies of gene BGL1 into the chromosome of *T. reesei* SEU-7 between KI911141.1:347357 and KI911141.1:347979 has caused a collateral mutation, the deletion of the original 623-bp fragment. Nevertheless, how this collateral mutation interferes other genes in SEU-7 is unknown, for the deleted gene sequence was not part of any open-reading frame (ORFs) of genes, whereas the mRNA level of its two adjacent genes M419DRAFT_127520 and M419DRAFT_6256 is under the limitation of detection by RT-qPCR. The missing of the original 623-bp fragment might take a part in the outperformance of SEU-7 at the chromatin-level regulation through chromatin remodeling as reported for the transcription factors involved in cellulase production. For example, both the transcriptional activator XYR1 and the transcriptional repressor CRE1 are involved in the chromatin remodeling. Chromatin rearrangement was observed in the XYR1 promoter during induction on sophorose, and the XYR1 promoter is overall more accessible in a truncated CRE1 background [39]. Also, CRE1 plays a key role in chromatin packaging under repression conditions [40–42]. CRE1 and the HAP2/3/5 complex take part in nucleosome assembly in the CBH2 promoter [42]. Furthermore, the insertion position probably affects the expression of the two inserted copies of BGL1 that is important as we have discussed above. Nevertheless, further investigations are required to explore these possibilities.

Transcription analysis of genes encoding major cellulases and the related genes with cellulase production were performed to identify key factors contributing to the excellent performance of SEU-7 over RUT-C30. Along with their enhanced protein expression and enzyme activities, significant upregulation was observed with the mRNA expressions of (hemi)cellulase genes. The MFS transporter CRT1 (tre3405) in *T. reesei*, which is considered as lactose permease [43, 44], is essential for cellulase production on cellulose, lactose, or sophorose, and is highly expressed during the growth of *T. reesei* on cellulose [45], lactose [45], and wheat straw [46]. These previous results well explain the remarkably elevated mRNA level of CRT1 we observed in SEU-7. It is reported that CRT1 was downregulated (78-fold) in the mutant strain *∆*XYR1 [47] when cellulose was used as the carbon source, suggesting a positive correlation between XYR1 and CRT1 occurs in the regulation of cellulase production. Consistently, the transcriptional level of XYR1 was unregulated in SEU-7 on cellulose, which was accompanied with the surged mRNA level of KAP8 that mediates the nuclear import of protein XYR1 [43]. Meanwhile, activator ACE3 displayed an enhanced mRNA level in SEU-7 on lactose, while activators ACE2 and HAP2/3 showed upregulated mRNA levels in SEU-7 on cellulose. On the contrary, the mRNA levels of genes participating in lactose metabolism were not changed noticeably in SEU-7. It seems that the increased transcription levels of cellulase genes, the related transcription factors, and the MFS transporter CRT1 contribute to the outstanding cellulase production of SEU-7.

Unlike model microorganisms *E. coli* and yeast, *T. reesei* does not possess well-developed genetic tools. Therefore, its engineering relies heavily on NTG mutagenesis, UV mutagenesis, and knockout or overexpression of a certain well-known gene to get mutant strains with desired performance, which definitely limits the ability to get *T. reesei* mutant strains with desired performance. Furthermore, the mechanism of cellulase production is unknown, preventing strain engineering with rational design. Thus, the random insertion method of AMT presents a useful strategy, as demonstrated here and in the previous studies [15, 48, 49], but has not been fully utilized for strain engineering in *T. reesei* and other fungi.

## Conclusions

It is the first time to obtain a *T. reesei* mutant SEU-7 that enables the soluble carbon source lactose as an efficient inducer as cellulose, presenting a substantial progress in the utilization of soluble carbon sources for cellulase production in *T. reesei*. In addition to the high yield, SEU-7 produced a complete whole set of cellulase and hemicellulase that are all improved notably on both cellulose and lactose, allowing a broad choice of pretreated biomass for saccharification. SEU-7 displays record FPase activity and BGL activity when grown on lactose. This outperformance of *T. reesei* SEU-7 is strain-specific as demonstrated by its different overexpression pattern of gene BGL1 from other recombinants, and is attributed to both the elevated expression level of gene BGL1 and the collateral mutation. Two copies of the inserted gene BGL1 were detected at the chromosome of *T. reesei* SEU-7 between KI911141.1:347357 and KI911141.1:347979, deleting the original 623-bp fragment that is not within any genes’ coding region. Unfortunately, it is still unclear how the identified collateral mutation gets involved in the excellent performance of SEU-7. Strain SEU-7 was altered significantly at gene transcription level as well as its phenotype like growth, extracellular glucose concentration, and protein secretion ability. *T. reesei* SEU-7 is definitely a better alternative to RUT-C30 in industry for cost-effective cellulase production using either lactose or cellulose.

## Additional file

**Additional file 1: Figure S1.** Schematic illustration of the plasmid pBGL. **Figure S2.** Cellulase activities of SEU-7 and RUT-C30 grown on different carbon sources. **Figure S3.** Effect of glucose on the enzyme activities of *T. reesei* RUT-C30 and SEU-7. **Figure S4.** The DNA sequence of the missing fragment at the loci of KI911141.1:351617-351930. **Table S1.** PCR primers for plasmid construction. **Table S2.** Primers for determination of copy numbers of BGL1 in SEU-7 using qPCR. **Table S3.** Primers for real-time quantitative PCR.

## Abbreviations

pNPGase: the β-glucosidase activity
pNPCase: the CBH activity
CMCase: the CMC activity
FPase: the filter paper activity
pNPXase: the β-xylosidase activity
AMT: agrobacterium tumefaciens-mediated transformation
PDA: potato dextrose agar
SDB: sabouraud dextrose broth
TMM: Trichoderma minimal medium
IU: international units
pNPG: *p*-nitrophenyl-β-d-glucopyranoside
EDA: ethylenediamine
PCS: the pretreated corn stover
DNS: 3,5-dinitrosalicylic
CCR: carbon catabolite repression
ORF: open-reading frame
DNS: 3,5-dinitrosalicylic
RT: reverse transcription
MFS: major facilitator superfamily sugar transporter
