# Supplementary material for: Cellulase hyper-production by Trichoderma reesei mutant SEU-7 on lactose
Source: Biotechnol Biofuels. 2017 Oct 4;10:228. doi: 10.1186/s13068-017-0915-9 (PMC5628480; doi:10.1186/s13068-017-0915-9)
Supplement: Supplementary file 1 — Additional file 1: Figure S1. Schematic illustration of the plasmid pBGL. Figure S2. Cellulase activities of SEU-7 and RUT-C30 grown on different carbon sources. Figure S3. Effect of glucose on the enzyme activities of T. reesei RUT-C30 and SEU-7. Figure S4. The DNA sequence of the missing fragment at the loci of KI911141.1:351617-351930. Table S1. PCR primers for plasmid construction. Table S2. Primers for determination of copy numbers of BGL1 in SEU-7 using qPCR. Table S3. Primers for real-time quantitative PCR. [file 13068_2017_915_MOESM1_ESM.docx]

**Additional file 1:**

**
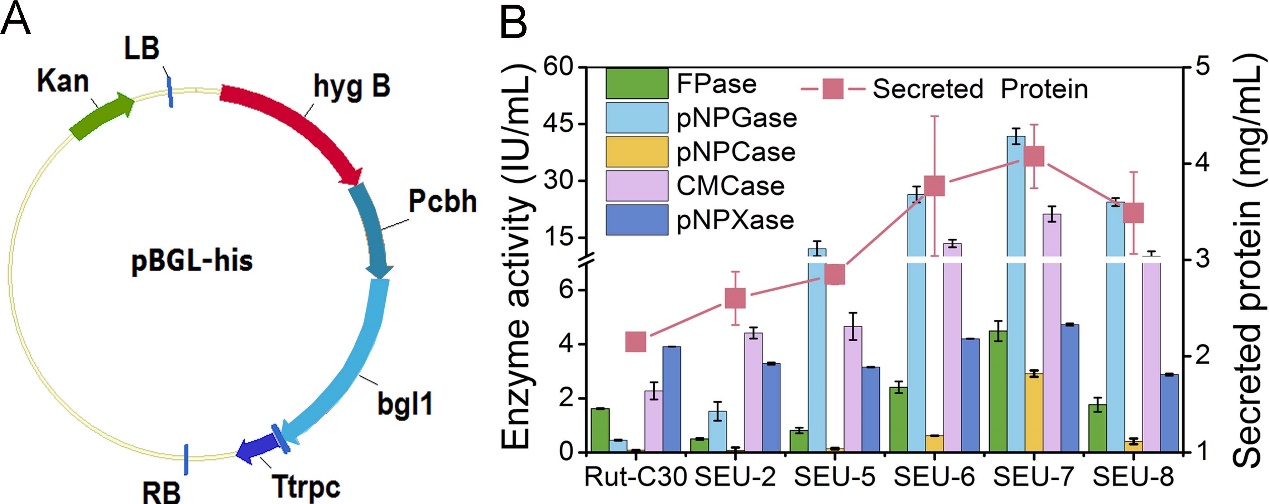
**

Fig. S1 Schematic illustration of the plasmid pBGL-his (A). Kan: kanamycin resistance; LB, left border of binary vector; RB, right border of binary vector; Pcbh, a modified CBH promoter [[15](#_ENREF_15)]; Ttrpc, *Aspergillus nidulans* trpC terminator; bgl1, *T. reesei* gene BGL1; hyg B, hygromycin B phosphotransferase gene. And the cellulase activities and secreted protein concentration of *T. reesei* RUT-C30 (day 5) and the five recombinant *T. reesei* strains: SEU-2, SEU-5, SEU-6, SEU-7, and SEU-8, using cellulose as the carbon source (B). pNPGase: the β-glucosidase activity; pNPCase: the CBH activity; CMCase: the CMC activity; FPase: the filter paper activity. Data are represented as the mean of three independent experiments and error bars express the standard deviations.

**
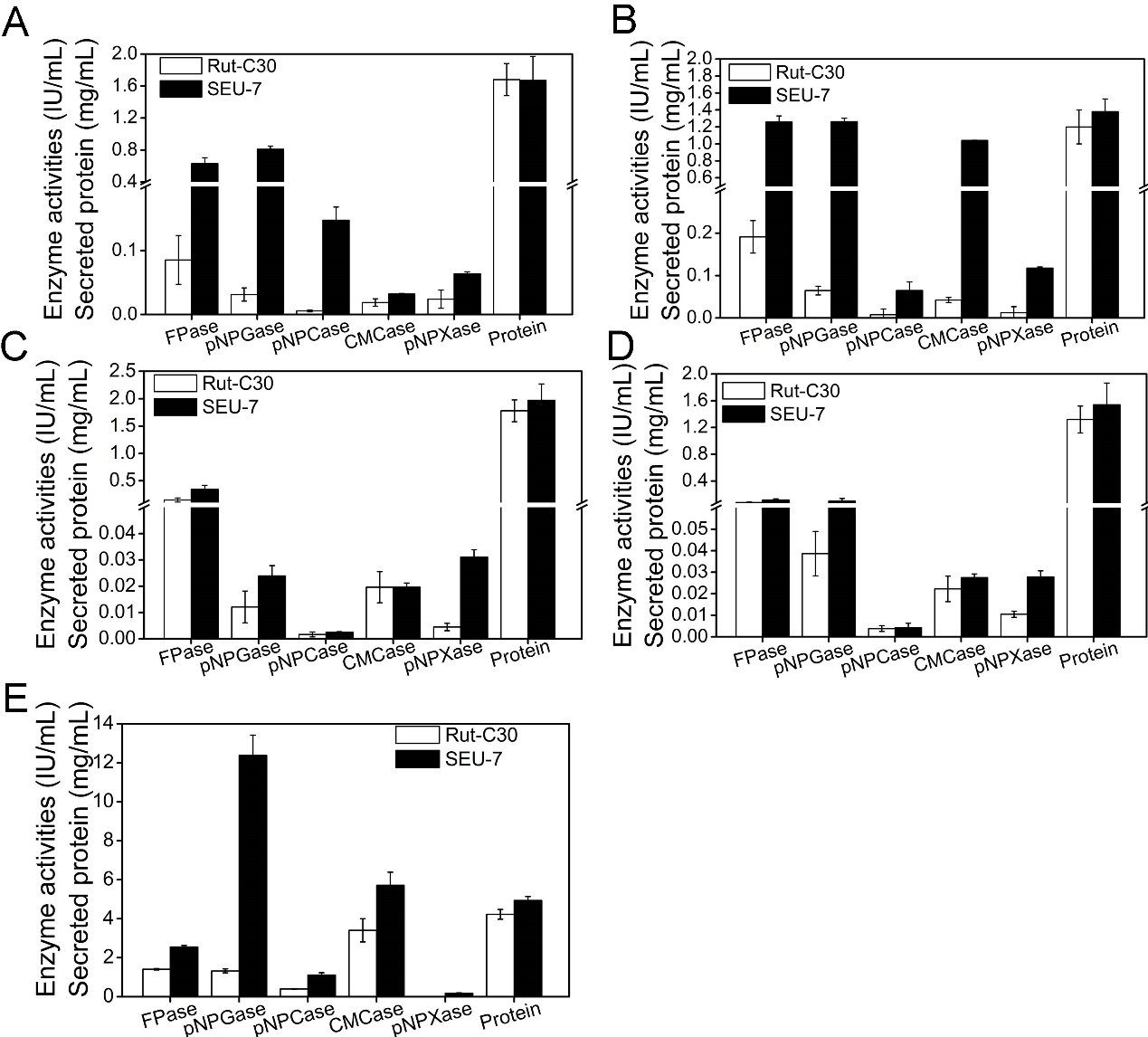
**Fig. S2 Cellulase activities of SEU-7 and Rut-C30 grown on glucose (A)，galactose (B)，sucrose (C), glycerol

(D) and cellobiose (E) for 5 days.
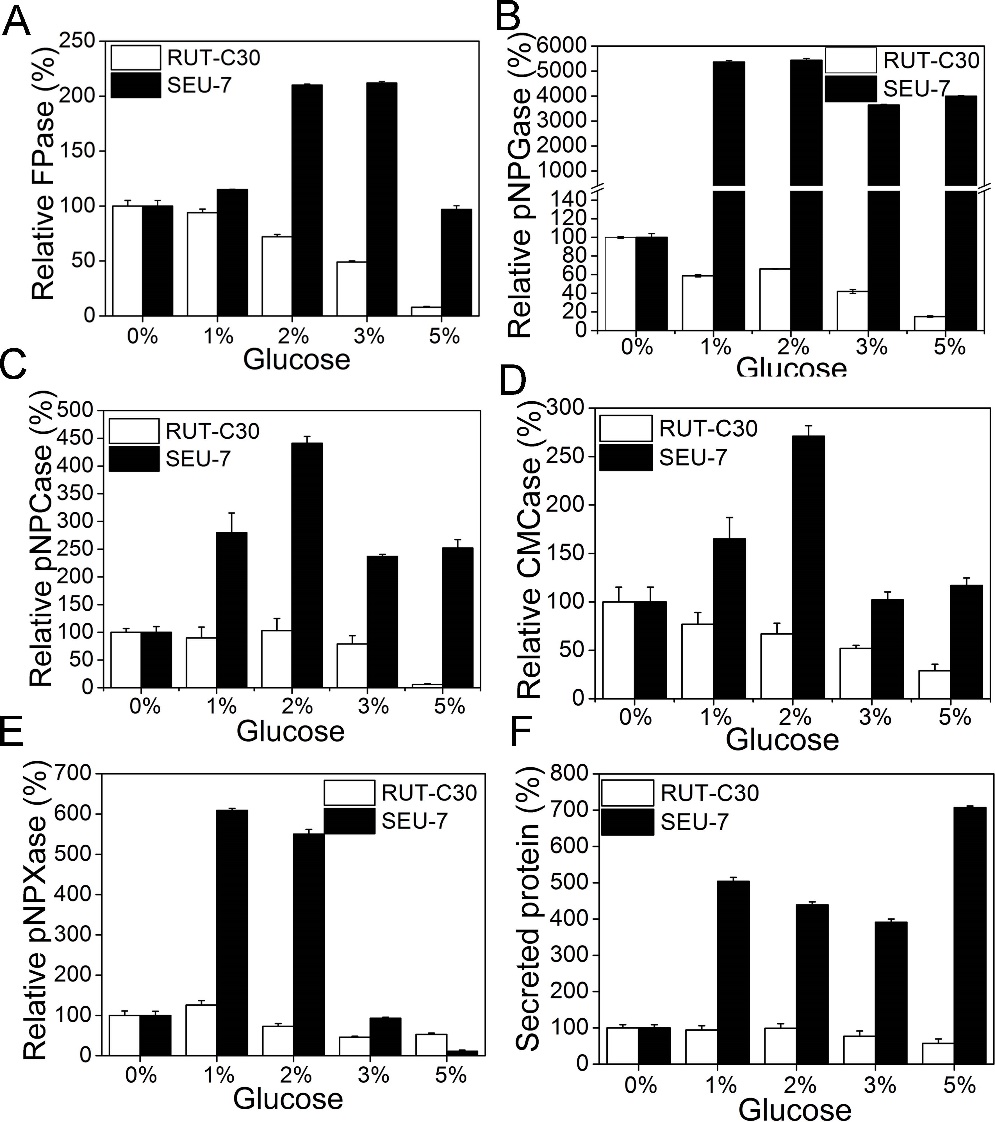


Fig. S3 Effect of 1%-5% glucose on the enzyme activities of *T. reesei* Rut-C30 and SEU-7 including the activity of pNPGase activity(A), pNPCase (B), FPase (C), CMCase (D), and pNPXase (E), and secreted protein concentration (F), when grown on 2% cellulose for 5 days. The error bars indicate the standard deviation of three biological replicates. The activities of pNPGase (BGL activity), pNPCase (CBH activity), FPA (Filter paper activity), CMCase (CMC activity) on day 5 in the absence of glucose are arbitrarily assigned as 100% in SEU-7 and Rut-C30 and SEU-7 individually and the corresponding activities are referred to Fig. 1.

GTCTTGGGATTTGTGTGTGGACGAGCTTGGACCAGGACCAGCATGAGGGGAGTTGGCCGT

CCTCGTAGGGCACGATGCGCGGTATGCGATATGCAATAATCGGGACGCAATGGGGGTGTC

GAGGCAAGCGGGATGAGCTCACCTACACGAGTACAAGCACCTATGAGAGGTAGGTACCTA

TGGAAACCGGAGCTCCATGATGTTATTTGTTCCCTGGGCTGATAGCCGGTGAAGAAGCTC

ACTGGGCTTTCATGGCAGTGTTGTAAAGGTATCTCATGCCATCCTGCCAAGTTGGCATCT

GATTTTGGATAGGAAATAGCGAGAGAGAGGTATCTGATCAATGGCATCATGACGGCACAC

TTCGACAATGAGGCTTACTAGGTACCTACTAAGGCAGACAACACAGCGCAACACAGTGTA

GCTGCTGCGCTCAAGCGACTTCGTAAGGCGCTTCTACCTTGCTGGCCCAGGCATATGAAG

CACACTTTATTCGATTTGTTGGACGAGAAGTGTCTGTCCGAGTATTCATACCTACCTACT

AGTAGGTAGGCACGATTTGGTACTATTGCGTGGGCTGCACTTCTTGCATGAGCTGCAGAT

TCTGCATATTGCCAAAGCCTCAT

Fig. S4. The DNA sequence of the missing fragment of 623 bp at the loci of KI911141.1：351617-351930.

Table S1 PCR primers for plasmid construction

| Primer | Sequence (5’to 3’) |
| --- | --- |
| BGL histaq Fw | ACCCAATAGTCAATCTAGAATGCGTTACCGAACAGCAGC |
| BGL histaq Rv | TCGGCATCTACTTCTAGATTAATGATGATGATGATGATGCGCTACCGACAGAGTGCTCG |

Table S2 Primers for determination of copy numbers of BGL1 in SEU-7 using qPCR

| Target gene | Forward primer(5’to 3’) | Reverse primer (5’to 3’) |
| --- | --- | --- |
| CEL3A-C-1 | GAGCAACCCAGATGACCGAA | AGCACATGACAGAAGCGACA |
| CEL3A-C-2 | TGCGGCCTTCGCCTTGTCGTAC | TTCGGTCATCTGGGTTGCTC |
| CEL3A-C-3 | GTGCCAACAGCAACGTCATT | TCATTGGGGCTCTTCGCAAT |
| CEL7A-C-1 | TACCTTATGGCGAGCGACAC | GCGTCAGGGGTTCATGGTAA |
| CEL7A-C-2 | GCCAGGTCCTGAACCCTTAC | CCAAGAATCTACCGGTGCGT |
| CEL7A-C-3 | ACTCCATCTCCGAGGCTCTT | CGTCTCGAACTGGGTGACAA |
| SAR1-C-1 | GCCGACTCTCCACCCTAGTA | GAAACCCTCCACAAACCCCA |
| SAR1-C-2 | CCGGAAAGACCACGTTGCTA | CCTCCTCCCATCGCAGAAAA |
| SAR1-C-3 | ATTCCCTTTCTCCGTTGCGG | CCGGATGTCGCATCAGGTAA |
| PGK1-C-1 | CCCCAAGTACTCCCTCAAGC | GACAATGGACTCGACCTCGG |
| PGK1-C-2 | AACCTCCGCTTCCACATTGA | GTAGATGTCGCCCAGAGCAG |
| PGK1-C-3 | TCATTATCGGCGGTGGTGAC | ACCTTTTCGCCATCTCTCCC |
| XYN3-C-1 | CTATGGCAAGGTCAACGGGT | GTGGCTCACCAATACCGTCA |
| XYN3-C-2 | TCTCGATTGCCTTTCGTGCT | CCCGTTGACCTTGCCATAGT |
| XYN3-C-3 | GTCTCGATTGCCTTTCGTGC | ACCCGTTGACCTTGCCATAG |

Table S3 Primers for real-time quantitative PCR

| Target gene | Forward primer(5’to 3’) | Reverse primer (5’to 3’) |
| --- | --- | --- |
| CEL3A | ATGCGTTACCGAACAGCAGCTGC | TGCGGCCTTCGCCTTGTCGTAC |
| CEL7A | GCGGATCCTCTTTCTCAGAC | TTGGCGTAGTAATCATCCCA |
| CEL7B | ACTACACGGAGGAGCTCGACGACTT | AAGGCATTGCGAGTAGTAGTCGTTG |
| XYN1 | CTCCGTCAACTGGTCCAACT | GTTGCCGTTTGGGTTGTAGC |
| XYN2 | TGACGTACACCAATGGTCCC | GTAGCTGCCCGAGAAGTTGA |
| XYN3 | GCAAGATTCGTGCTTGGGAC | ATCGAGACAAACTCCTCGCC |
| BXL | CGAGTTTGGCAGTGGTCTCT | TGTGCGAACAAACAGCATGG |
| VEL1 | ATCGCGTGACGAGAGAGAAC | ATGCAGGAACACCAGTCAGG |
| ACE1 | AAGACCCTGATCTTCATGGC | ATTCGACTGTCGCTTGAATG |
| ACE2 | GCAGCAGCTGAGAGAGTACG | ATAGAGGGAGGCGAGATCCT |
| ACE3 | CCCAAGTACTCGTGGCACAT | ATGGTGATGGGCCGATTGTT |
| KAP8 | ATCTTGGCGAGGACGATGAC | GGTCTGCTCTCCAGCAACAT |
| HAP2 | CGTAACGCCAAGCTGGTCTA | GGCACGAAACAGTCTCACCT |
| HAP3 | CAAGACCGGTGGTTGCCTAT | AACTCGCTCACACACTCCTG |
| CRT1 | CCTTTTCCAGCTTTGCCACC | CTCTTCCAAAGTGCGTCCCT |
| LAE1 | GGTTCTTGGAAGCCTGGGAA | TCGGCATCCTTGGCATACTC |
| XYR1 | TACCAAGTGCGATGGCTTAC | CTCTCTCGGACATATTCGCA |
| LAD1 | GCCATCTCAGTCAAGCCCAA | GCGTGCCAGAAATGGACATC |
| LXR | CCGCCAACAGACTCCTTGAT | GCGAGACGAGTAGGTGATGG |
| HXK | CTCGCCAAAGGTCTCAGTGT | TCCGTCAAGGTGATTTCGCA |
| GAL1 | GCGCAATCATTCGTCACCTC | CTCCTTGGTGAGGCTCTTGG |
| GAL7 | AATTTCCCCGCAAAACCGTC | GTGAGTGGGTTGTACCTCCG |
| GAL10 | AAGCCTTTCGAGCAGTGGAA | GCAGCAGGTTGAAAGGAACG |
| CRE1 | GAGGACCACCGCATACTAGC | GAGCGGCAGTCAAAAAGCAA |
